# Aging Impairs Disengagement From Negative Words in a Dot Probe Task

**DOI:** 10.3389/fpsyg.2018.02361

**Published:** 2018-11-28

**Authors:** Christine E. Talbot, John C. Ksander, Angela Gutchess

**Affiliations:** Aging, Culture, and Cognition Laboratory, Department of Psychology, Brandeis University, Waltham, MA, United States

**Keywords:** aging, emotion, dot probe, attention, disengaging, orienting

## Abstract

Age differences in emotional processes have been of great interest. Previous studies using the dot probe task show that older adults can be more influenced by negative emotionally valenced faces than younger adults. Subsequent work has demonstrated two distinctive ways people engage with stimuli in this task, namely orienting to and disengaging from emotional stimuli. In the present study, we examined the effects of aging as well as ability to orient to and disengage from emotional words in a dot probe task. Older and younger adults viewed word pairs (positive-neutral, negative-neutral, and neutral-neutral) on a computer screen and pressed a button to identify a probe that replaced one of the words in the pair, responding as quickly as possible. Probes replaced either the emotional or neutral word. This design tests whether effects of aging were larger for disengaging (identifying a probe that replaced a neutral word in an emotional-neutral trial), compared to orienting (identifying a probe that replaced an emotional word in an emotional-neutral trial), and whether the pattern was exaggerated for negative compared to positive stimuli. Attentional bias estimates were calculated with mean reaction times for each trial-type. Older adults showed a specific impairment in disengaging from negative words. These results could reflect challenges with cognitive control and inhibition with age, which in this study are larger for older adults in the presence of negative information.

## Introduction

Many aspects of cognition decline with age. However, some aspects of socioemotional processing may be preserved with the potential to bolster cognition (Kensinger and Gutchess, 2017). Moreover, age groups may differ in their prioritization of emotional information, such as older adults avoiding negative information to a greater extent than young (Mather and Carstensen, 2003).

To understand how emotion influences attention with age, some research employs the dot probe task. With this paradigm, older adults respond more quickly to positive than neutral faces, and more slowly to negative faces than neutral faces; younger adults are influenced little by emotional faces (Mather and Carstensen, 2003). Converging with these results, older adults demonstrate an avoidant-vigilant response to angry faces, while younger adults are less affected by angry faces (Lee and Knight, 2009). Other variations of age differences in attention biases have emerged, such as age differences in attentional orienting emerging only for positive-negative pairs, but not neutral-negative pairs, such that younger adults show a bias toward negative faces whereas older adults do not (Tomaszczyk and Fernandes, 2014). Eye-tracking reveals that older adults look away from sad and toward happy faces more than younger adults, though older adults tend to respond more quickly to probes replacing happy faces rather than sad faces in the dot probe task (Isaacowitz et al., 2006a). Aside from the dot probe paradigm, eye-tracking measures show that older adults fixate less frequently on negative pictures than younger adults, while still reporting higher levels of negative emotions (Wirth et al., 2017). Older adults also self-report greater levels of sadness than young while watching sad film clips (Kunzmann and Grühn, 2005), suggesting that older adults process valenced stimuli differently than young, though it remains unclear what role attention plays in these differences.

Although it is evident that there are age differences in attention and processing of emotional information, it is unknown which components of attention differ with age. The dot probe task has the potential to distinguish the processes of orienting to and disengaging from emotional stimuli (Salemink et al., 2007), which allows for identification of which processes are affected by aging. Age differences in orienting could indicate that older adults seek out more positive information in their environments, whereas age differences in disengagement could indicate that older adults experience more difficulty disengaging from negative emotional information once it is encountered, perhaps due to cognitive control impairments.

Aging research thus far has not distinguished between orienting and disengaging processes in dot probe tasks, although other studies have found such an approach illuminating. Compared with low anxious individuals, highly anxious younger adults show impairments in disengaging from negative words, but do not differ in orienting to negative words (Salemink et al., 2007). Another non-clinical population exhibited the same pattern; participants had difficulty disengaging from mildly and highly threatening pictures compared to neutral, but were no quicker orienting to threatening than neutral stimuli (Koster et al., 2004). These results suggest that orienting to emotional stimuli remains consistent for young adults in Western societies; impairments in disengaging drive differences in attentional bias.

As prior work reveals larger effects for disengagement from emotional information rather than orienting, we predict this will also be the case for aging. Such a pattern would converge with a large literature indicating age impairments in inhibition or cognitive control (e.g., Hasher and Zacks, 1988; Kray and Lindenberger, 2000; Amer et al., 2016; but see Rey-Mermet and Gade, 2018 which challenges this notion), processes that would be necessary to support disengagement from negative emotional information. The present study addresses this gap in the literature.

## Methods

Fifty-eight younger [*M*_age_ = 19.06 (*SD* = 1.42); *M*_yearsofeducation_ = 12.81 (*SD* = 1.71)] and 48 older adults [*M*_age_ = 75.84 (*SD* = 7.42); *M*_education_ = 16.34 (*SD* = 2.48); *M*_MMSE_ = 28.43 (*SD* = 1.82)] participated, for credit (majority of young) or pay (remaining participants). The Brandeis IRB approved the study; participants provided written informed consent.

In the dot probe paradigm, two stimuli flash simultaneously on opposite sides of a computer screen. On some trials, a dot (the probe) appears in the location of one of the previously presented stimuli. Attentional bias can be measured by subtracting the mean reaction time (RT) to probes replacing threatening stimuli from the mean RT to probes replacing neutral stimuli in threat-neutral pairs (Mogg et al., 2000; Mather and Carstensen, 2003). High attentional bias indices could indicate low RTs to threat stimuli (reflecting quick orienting to threatening stimuli), high RTs to neutral stimuli (reflecting difficulty disengaging from threatening stimuli), or a combination of these two (Salemink et al., 2007).

Stimuli were selected by sampling subsets of word pairs from a database (Warriner et al., 2013). First, samples comprising 32 positive-negative pairs were iteratively drawn from the database while maximizing the similarity of each positive-negative pairing on six dimensions: absolute valence, arousal, frequency, number of syllables, number of letters, and orthographic neighborhood size. This ensured pairs were individually well-matched on two emotional and four lexical properties. Second, emotional words were paired with neutral words from the database following the same procedure, yielding positive-neutral and negative-neutral word pairs with well-matched characteristics. Finally, neutral-neutral word pairs were selected with the same method. This iterative optimization procedure was implemented with custom MATLAB code. There were 128 total word pairs (32 negative-neutral, 32 positive-neutral, and 64 neutral-neutral pairs, 32 serving as fillers for which no probe appeared) presented in one block in a random order unique to each participant adapted from Salemink et al. (2007).

Trials began with a 500 ms fixation cross, followed by a word pair for 500 ms. Words were presented on the screen one above the other, separated by 3 cm. After the words disappeared, an asterisk replaced the location of one of the words. Probes were either congruent (replacing emotional-word) or incongruent (replacing neutral-word), appearing equally often at the top or bottom location. Participants pressed a key as quickly as possible to indicate if the asterisk was in the top or bottom location.

## Results

Participants were excluded for inattentiveness (one young), inability to perform tasks (two older), and MMSE scores ≤ 26 (seven older). Young and older adults completed ≥ 50% of each trial type; outlier RTs (< 200 ms, > 2000ms, or 2.5 *SD*s +/- each participant’s mean) were excluded. See Table 1 for mean RTs.

**Table 1:** Mean RTs (ms) of congruent, incongruent, and neutral trials.

Condition	Age	Mean RT	Standard deviation
RT Positive congruent	Young	444.02	61.79
	Old	658.23	114.30
RT Negative congruent	Young	443.83	55.66
	Old	664.12	121.42
RT Neutral	Young	445.68	55.26
	Old	661.96	123.08
RT Positive incongruent	Young	438.36	48.29
	Old	656.48	123.44
RT Negative incongruent	Young	443.24	56.97
	Old	679.56	135.82


To examine specific attention processes, neutral-neutral word pair trials were included to calculate attentional bias indices for each participant (Salemink et al., 2007). The orienting index, identifying probes in congruent trials can be calculated as:

Orienting Index=Mean RT Neutral−Mean RT Emotional

The disengaging index, identifying probes in incongruent trials can be calculated as:

Disengaging Index=Mean RT Emotional−Mean RT Neutral

Separate orienting and disengaging indices were calculated for positive and negative trials.

**FIGURE 1:**
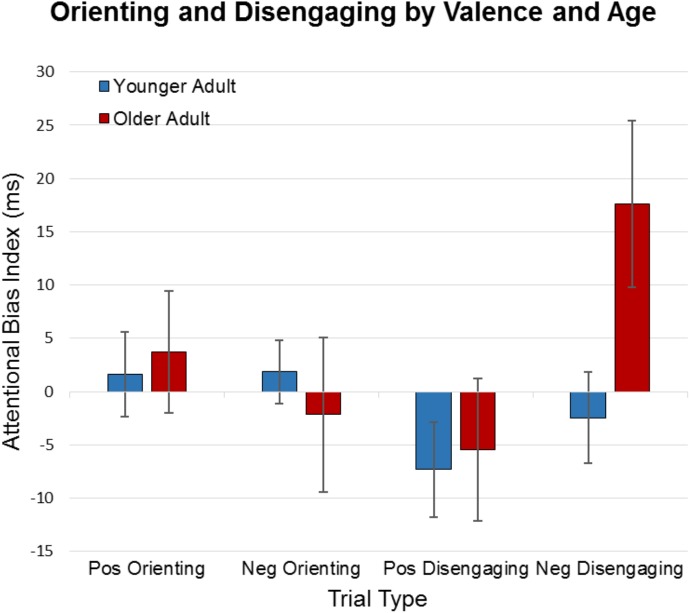
Attentional bias indices from the dot probe task. Attentional bias indices were calculated for each condition; a mean score of zero indicates no attentional bias. Older adults demonstrated the greatest impairment in disengaging from negative words compared with positive words, while negative and positive words did not differ for orienting. No significant differences in performance were found in younger adults.

Using these indices, we conducted a 2 × 2 × 2 ANOVA to examine the effects of age (young/old), as a between-participants variable, and valence (positive/negative) and detection (orienting/disengaging) as within-participant variables on reaction times. Critically, results revealed a significant three-way interaction between age × valence × detection, *F*(1,104) = 4.05, *p* = 0.05, η_p_^2^ = 0.04. There was also a valence × detection interaction, *F*(1,104) = 7.78, *p* = 0.01, η_p_^2^ = 0.07. We found a marginal main effect of valence, *F*(1,104) = 3.67, *p* = 0.06, η_p_^2^ = 0.03, but no other main effects or interactions approached significance (η_p_^2^ ≤ 0.02). Average attentional bias scores for younger and older adults appear in Figure 1. To further examine age differences in the effects of valence (positive/negative) and detection (orienting/disengaging), additional 2 × 2 ANOVAs were conducted separately for each age group. The interaction of valence × detection was significant in older adults, *F*(1,47) = 6.08, *p* = 0.02, η_p_^2^ = 0.11, but did not approach significance in younger adults, *F*(1,57) = 0.85, *p* = 0.37, η_p_^2^ = 0.02. Older adults took considerably longer to disengage from negative words than positive words, *t*(47) = 2.98, *p* = 0.005, but negative and positive words did not differ for orienting (*p* = 0.43). In contrast, there were no significant differences for younger adults, *p*s > 0.26.

Additional analyses allowed for comparison with prior studies (Mather and Carstensen, 2003; Isaacowitz et al., 2006b). Based on those studies, we calculated attentional bias scores by subtracting congruent from incongruent RTs for positive and negative trials. A 2 × 2 ANOVA with age (young/old) and valence (positive/negative) revealed a marginal effect of valence, with higher bias scores for negative trials (indicating longer RTs when probes replaced the negative than the neutral item in the pair) than positive trials, *F*(1,104) = 3.67, *p* = 0.06, η_p_^2^ = 0.03. The effect of age and its interaction with valence were not significant (η_p_^2^ = 0.01). Our results contrast prior studies finding biases toward positive and away from negative stimuli for older adults.

## Discussion

Distinguishing between orienting and disengaging to emotional stimuli in the dot probe task is revealing in illustrating which components of attention differ across groups. To summarize our results, older adults exhibit a reduced ability to disengage from negative words, but not positive words. Older adults are not biased in orienting to either negative or positive words, and younger adults show no differences in orienting or disengaging in the presence of positive and negative words.

Our findings suggest that older adults are more influenced by emotional stimuli in dot probe tasks, whereas younger adults are less affected (Mather and Carstensen, 2003; Lee and Knight, 2009; but see Tomaszczyk and Fernandes, 2014). Although this conclusion is overall in line with prior studies (using faces rather than words), we relied on orienting and disengaging measures and comparisons with control (neutral-neutral) trials (i.e., we did not replicate effects using attentional bias calculations from prior studies). Disengagement and orienting measures allow for consideration of more specific processes. In finding that older adults have the greatest impairment in disengaging from negative words compared to positive words, the results distinguish for the first time the effect of aging on dot probe measures of disengaging and orienting. This impairment in disengaging from negative stimuli is consistent with other work showing that both high- and low-anxiety younger adults have difficulty disengaging from negative stimuli (Koster et al., 2004; Salemink et al., 2007). In addition, our results demonstrated that younger adults did not show any differences in orienting or disengaging to negative information, in line with much of the research indicating larger differences for older than younger adults as a function of emotion. Interestingly, some other paradigms reveal that clinical populations, such as individuals with major depression or anxiety, are quicker to orient to negative stimuli, and show no differences in disengaging, compared to non-clinical groups (Gotlib et al., 2004; Klumpp and Amir, 2009).

Two potential limitations are present in this study. First, we did not manipulate the stimulus onset asynchrony (SOA) in our task design. Other work has demonstrated that SOA can critically impact the reliability of the attentional bias scores on the dot probe task for younger adults (Chapman et al., 2017). Second, age differences in emotion may not extend across cultures, based on some research suggesting cultural differences in positivity effects (Fung et al., 2008; but see Kwon et al., 2009). Future work should address these limitations.

In terms of broader implications, older adults’ impairments in disengaging from negative words could impact well-being. Greater difficulty disengaging attention from negative information can result in negative emotional states, such as prolonged periods of anxiety (Koster et al., 2004; Salemink et al., 2007). Given that older adults generally have strong emotion regulation abilities, it may be surprising that they are more captured by negative information than younger adults. However, performance on this task likely differs from real-world emotion regulation situations in which older adults can adopt different strategies to regulate emotion, such as limiting exposure. For example, older adults may use situation selection or look away from negative information, as shown in eye-tracking studies (Isaacowitz et al., 2006b). When older adults fail to regulate initial exposure to information, they may need to engage more reactive control to disengage from information, whereas younger adults can engage cognitive control processes at the front end, thus preventing effortful disengagement after exposure (Velanova et al., 2007; Braver et al., 2009). As some other tasks show that older adults can be immune from interference on some emotional tasks (Samanez-Larkin et al., 2009), the ways in which emotion and control processes interact will continue to be of interest in future research.

## Data Availability Statement

The raw data supporting the conclusions of this manuscript are available through the Open Science Framework: https://osf.io/mzcgk/?view_only=5a1eabae580b43b8b76d440f49f3b98e

## Author Contributions

CT, JK, and AG contributed conception, design, and framing of the study. CT and JK collected data. CT and JK organized data. CT and AG performed the statistical analysis. CT wrote the first draft of the manuscript. All authors contributed to manuscript revision, and read and approved the submitted version.

## Conflict of Interest Statement

The authors declare that the research was conducted in the absence of any commercial or financial relationships that could be construed as a potential conflict of interest.
